# Supplementary material for: Complex‐centric proteome profiling by SEC‐SWATH‐MS
Source: Mol Syst Biol. 2019 Jan 14;15(1):e8438. doi: 10.15252/msb.20188438 (PMC6346213; doi:10.15252/msb.20188438)
Supplement: Supplementary file 7 — Dataset EV6 [file MSB-15-e8438-s007.zip › feature_plots_bioplex/P51970.pdf]

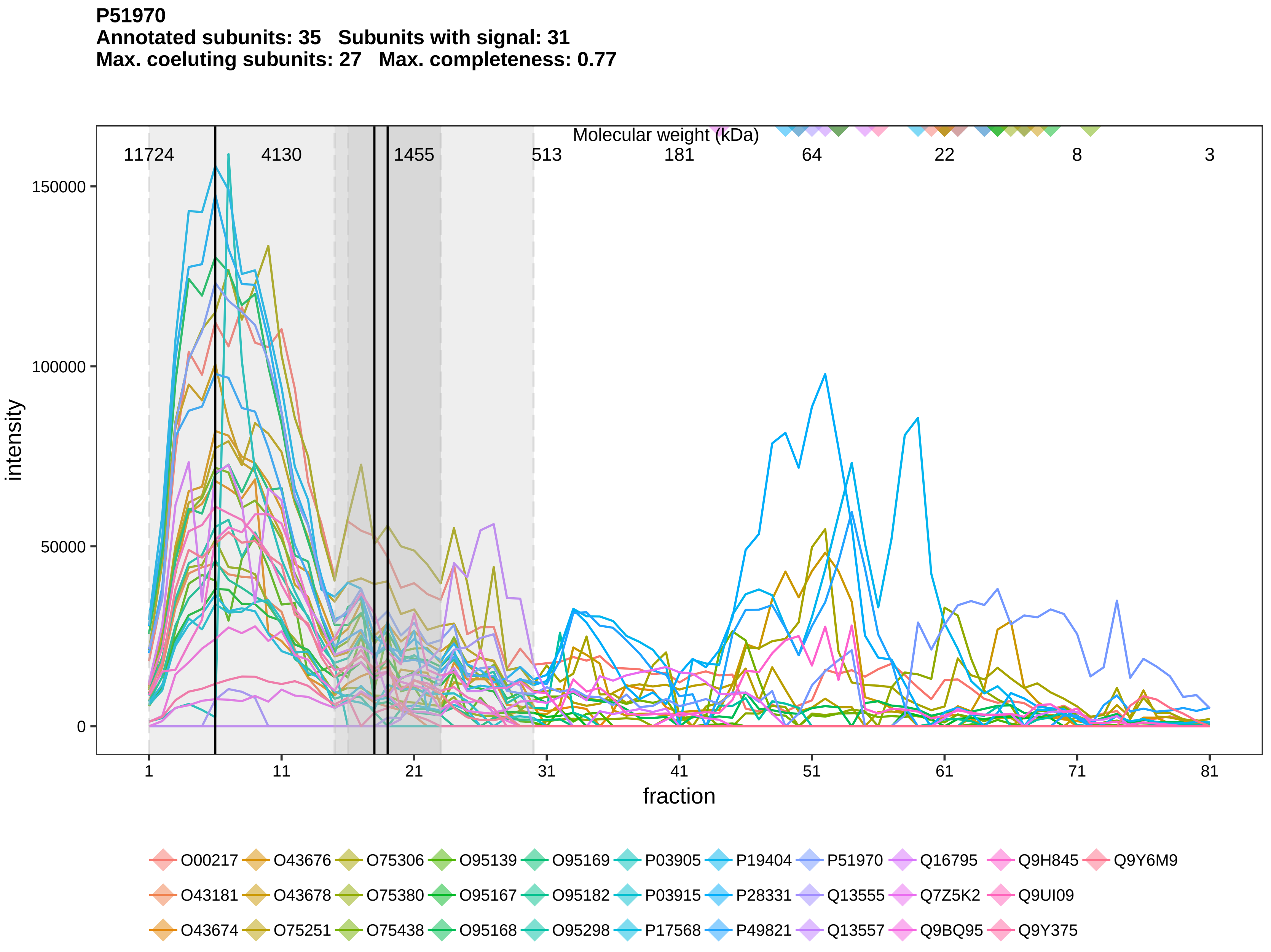

P51970
Annotated subunits: 35 Subunits with signal: 31
Max. coeluting subunits: 27 Max. completeness: 0.77
Molecular weight (kDa)
11724
4130
1455
513
181
64
22
8
3
150000
100000
50000
0
intensity
1
11
21
31
41
51
61
71
81
fraction
O00217
O43676
O75306
O95139
O95169
P03905
P19404
P51970
Q16795
Q9H845
Q9Y6M9
O43181
O43678
O75380
O95167
O95182
P03915
P28331
Q13555
Q7Z5K2
Q9UI09
O43674
O75251
O75438
O95168
O95298
P17568
P49821
Q13557
Q9BQ95
Q9Y375
